# Glossoptosis After Tracheal Extubation in a Patient With Oculopharyngeal Muscular Dystrophy: A Case Report

**DOI:** 10.7759/cureus.93696

**Published:** 2025-10-02

**Authors:** Chihiro Akizawa, Joho Tokumine, Hironori Motoyama, Noriko Takeuchi, Moeko Okuni, Tomoko Yorozu, Kiyoshi Moriyama

**Affiliations:** 1 Anesthesiology, Kyorin University School of Medicine, Mitaka, JPN

**Keywords:** glossoptosis, muscle relaxant monitor, oculopharyngeal muscular dystrophy, rocuronium, sugammadex, train-of-four ratio

## Abstract

Oculopharyngeal muscular dystrophy is a late-onset genetic disorder that causes progressive weakness of the eyelid and pharyngeal muscles. Herein, we report a case of oculopharyngeal muscular dystrophy and glossoptosis following extubation.

A 53-year-old Japanese woman with oculopharyngeal muscular dystrophy was scheduled to undergo surgery for ptosis. Although dysphagia was not observed, she experienced mild limb numbness. Total intravenous anesthesia was administered, followed by intravenous rocuronium (0.5 mg/kg), and tracheal intubation was performed. The train-of-four ratio exceeded 1 before surgery was completed. After surgery, the patient was awakened and extubated. However, glossoptosis and hypoxia developed, necessitating sugammadex administration.

In oculopharyngeal muscular dystrophy, even patients without apparent pharyngeal or laryngeal symptoms may have impaired upper airway muscle function. Complete recovery of the adductor pollicis train-of-four ratio does not necessarily ensure safe extubation. Since current guidelines provide no recommendations for such cases, further discussion and the development of management strategies, including the consideration of routine reversal, are warranted.

## Introduction

Oculopharyngeal muscular dystrophy (OPMD) is a rare, late-onset form of muscular dystrophy that usually begins after the age of 40 years [1,2]. Affected individuals often develop eyelid muscle weakness first, which leads to ptosis that gradually worsens and may result in decreased vision or limited eye movement [1]. Pharyngeal muscle weakness can also occur, causing dysphagia and dysphonia; however, the respiratory and cardiac muscles are generally spared, and the overall prognosis is relatively favorable [1].

OPMD is most commonly inherited in an autosomal dominant manner, although autosomal recessive cases have been described [2]. Its prevalence in France is estimated at approximately one in 100,000 individuals; the worldwide prevalence remains uncertain [2]. The disease results from an abnormal expansion of trinucleotide repeats in the poly(A)-binding protein nuclear 1 gene [2].

Herein, we report the case of a patient with OPMD who developed glossoptosis after extubation despite an apparent recovery of the train-of-four (TOF) ratio and required administration of a neuromuscular blockade reversal agent.

## Case presentation

A 53-year-old woman (height, 157 cm; weight, 70 kg) was scheduled to undergo surgery for ptosis due to muscular dystrophy. The patient was diagnosed with OPMD. Aside from ptosis, her only symptoms were mild limb numbness and difficulty in walking long distances. She did not have dysphagia or phonatory symptoms. The onset of ptosis was unknown; further, at the age of 47 years, when the patient underwent hysterectomy and oophorectomy, she did not have any symptoms related to muscular dystrophy. Her medical history included atrial fibrillation, for which she successfully underwent radiofrequency catheter ablation. Her younger brother was also diagnosed with OPMD. The laboratory data revealed mildly elevated liver transaminase levels and a decreased estimated glomerular filtration rate (Table 1).

**Table 1:** Laboratory findings on admission

Laboratory test	Result	Reference range	Unit
Aspartate aminotransferase	45	13–30	IU/L
Alanine aminotransferase	55	7–23	IU/L
Gamma-glutamyl transpeptidase	54	9–32	IU/L
Alkaline phosphatase	73	35–61	IU/L
Estimated glomerular filtration rate	65.7	>90	mL/min

**Figure 1:**
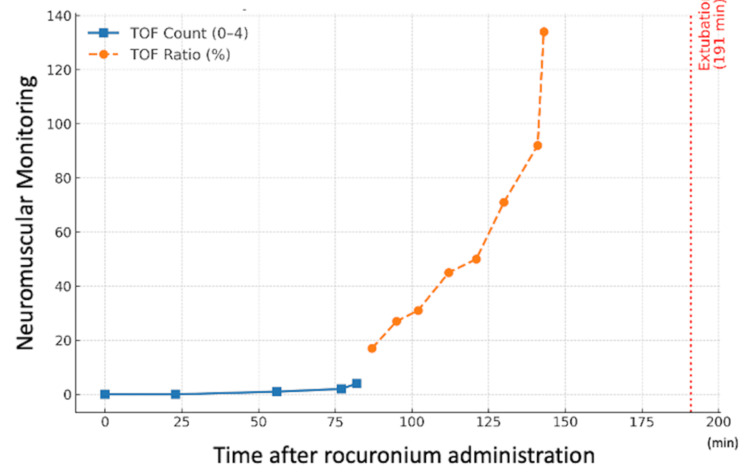
Neuromuscular monitoring after rocuronium administration This figure shows the time course of neuromuscular monitoring after neuromuscular blocking agent (rocuronium) administration. The train-of-four (TOF) count decreased to zero and gradually recovered over time. The TOF count (blue squares, solid line) was monitored until the count value reached 4, after which the TOF ratio (orange circles, dashed line) was measured. The TOF ratio reached 100% (overshot) at 143 minutes after neuromuscular blocker administration. Extubation was performed at 191 minutes (red dotted line).

The preoperative evaluation indicated a small chin, short neck, inter-incisor distance of two fingerbreadths (Mallampati class III), and upper lip bite test class III, predicting difficult intubation. Considering the risk of rhabdomyolysis, total intravenous anesthesia was selected. Upon entering the operating room, standard monitoring (electrocardiogram, saturation of percutaneous oxygen (SpO2), non-invasive blood pressure) was applied, along with an acceleromyography-based neuromuscular monitor (TOF-Watch®, Tokyo, Japan) on the forearm (measured from the adductor pollicis muscle) and a bispectral index monitor on the forehead. After preoxygenation (6 L/min for three minutes), general anesthesia was induced with fentanyl (100 μg intravenous injection (IV)), remifentanil (0.1 μg/kg/min infusion), ketamine (20 mg IV), and propofol (target-controlled infusion (TCI), target concentration: 3.0 μg/mL). The patient lost consciousness at a predicted effect-site concentration of 0.8 μg/mL, after which neuromuscular monitoring was calibrated. Mask ventilation was mildly difficult and improved after rocuronium administration (35 mg, 0.5 mg/kg IV). Tracheal intubation was performed using a McGRATH™ with Blade Size 3 (Medtronic Japan Co., Ltd., Tokyo, Japan) and a Ring-Adair-Elwyn tube (inner diameter 7.0 mm) at a TOF count of 0. Surgery comprised a frontalis sling procedure using autologous fascia lata from the thigh. The total intraoperative fentanyl dose was 300 μg, administered based on pharmacokinetic predictions (Shafer’s 3-compartment model). The bispectral index values were maintained at 40-60 during surgery. Rocuronium was administered once at induction (35 mg, 0.5 mg/kg) without additional doses. At 143 minutes post-administration, the TOF ratio exceeded 100% (during supramaximal calibration, subsequent measurements before intubation, and those performed when the TOF ratio exceeded 100% we ensured consistent positioning of the forearm and hand) (Figure 1). The surgery lasted 129 minutes. 

After surgery, the continuous infusion of propofol and remifentanil was terminated. At a TCI predicted effect-site propofol concentration of 1.2 μg/mL, the patient regained consciousness and responded to commands verified by hand squeezing (the predicted effect-site fentanyl concentration was 0.7 ng/mL). Prior to extubation, the patient was alert and oriented, with spontaneous ventilation (approximately 4 L/min), SpO₂ 99% at a fraction of inspired oxygen 0.4, stable hemodynamics, and normothermia of 36.5 °C. These conditions met standard extubation criteria. The tracheal tube was removed after oral suction (Figure 1). The patient had difficulty expectorating oral secretions and speaking. Paradoxical breathing was observed with a gradual decrease in SpO2 from 100% to 84%. Mask ventilation with 100% oxygen at 8 L/min was initiated because glossoptosis was considered the cause. SpO2 improved to 93%, although glossoptosis did not improve; therefore, sugammadex (100 mg IV) was administered. However, there was no improvement, and an additional 100 mg of sugammadex was administered two minutes later, after which glossoptosis resolved, gradually leading to an SpO2 recovery of 97%. The patient was switched to oxygen therapy via a facemask, and SpO2 further improved to 99%. The total anesthesia duration was 210 min. The patient was then transferred to the surgical intensive care unit for postoperative monitoring. 

Postoperatively, the patient’s hoarseness resolved by the next day, and she experienced no further laryngopharyngeal symptoms. She was subsequently discharged without complications.

## Discussion

Residual effects of anesthetics, such as propofol or fentanyl, may contribute to upper airway obstruction. However, based on the pharmacokinetic modeling of these agents and this patient’s level of consciousness, spontaneous respiratory effort, and predicted effect-site concentrations at the time of extubation, we considered significant sedation-related respiratory depression unlikely. Therefore, a more plausible interpretation is that a minimal but clinically relevant amount of neuromuscular blockade persisted, which was sufficient to compromise upper airway patency despite apparent full recovery at the monitored site. Acetaminophen is typically administered for this surgery; however, it was avoided for this patient due to mild hepatic impairment. Instead, a small dose of ketamine was administered during the two-to-three-hour surgery, which was unlikely to affect consciousness while providing supplemental analgesia. 

Roma Amyot, who was instrumental in establishing OPMD as a distinct clinical entity, studied 10 families with the disease and found that approximately 60% of patients with ptosis did not exhibit pharyngeal symptoms [3]. Our patient, who had ptosis but no dysphagia, exhibited similar findings. Genotype-phenotype correlations may explain the variability in symptom presentation [1]. Although the severity of ptosis and dysphagia may vary, both are expected to eventually manifest. For instance, OPMD caused by glycine-cysteine-asparagine heterozygosity is reported to fully develop by the age of 70 years [1]. In a study by O’Laughlin et al. [4], esophageal motility abnormalities were found in the asymptomatic siblings of patients with OPMD, suggesting that pharyngeal dysfunction may be present even in the absence of subjective symptoms. 

Landrum and Eggers [5] reported the first case of general anesthesia in a patient with OPMD. They monitored the masseter muscle and administered neostigmine and glycopyrrolate after a TOF count of 4 in that muscle, assuming that it more accurately reflected pharyngeal muscle recovery. This suggests that selecting an appropriate monitoring site is crucial when managing patients with neuromuscular diseases. As the surgical field involved the eyelid, monitoring the orbicularis oculi muscle [6] was not feasible in this patient. Nonetheless, the use of neuromuscular blocking agents during general anesthesia requires quantitative neuromuscular monitoring and appropriate reversal to prevent postoperative respiratory complications [7]. Unfortunately, current guidelines do not offer specific recommendations for patients with underlying neuromuscular disorders [7]. A systematic review by Gurunathan et al. [8] summarized case reports that demonstrated successful reversal of rocuronium with sugammadex in patients with neuromuscular disorders. However, adverse events, including anaphylaxis and incomplete reversal, have also been reported. The authors emphasized the importance of using quantitative neuromuscular monitoring to confirm adequate recovery in these vulnerable populations [8]. Since the introduction of sugammadex, virtually no reports exist of general anesthesia with rocuronium in patients with Duchenne muscular dystrophy without the use of sugammadex [9]. Although quantitative monitoring is recommended in neuromuscular disorders, no specific guidelines currently address sugammadex use when the TOF ratio has already exceeded 100%. Although sugammadex can be effective, the risk of adverse events such as anaphylaxis must also be considered, necessitating careful individualized decisions.

## Conclusions

In summary, our patient with OPMD did not have apparent pharyngeal or laryngeal symptoms and a TOF ratio >100%, but nevertheless developed glossoptosis after extubation. This suggests subclinical involvement of the pharyngeal muscles despite apparently normal neuromuscular monitoring. Since current guidelines do not provide recommendations for such cases, further discussion and the development of management strategies, including consideration of routine reversal, are warranted. Moreover, although the patient fulfilled standard extubation criteria, including adequate spontaneous ventilation, stable oxygenation and hemodynamics, and normothermia, tongue protrusion and head lift were not assessed. This case highlights that subclinical laryngopharyngeal dysfunction may occur even without overt symptoms, underscoring the importance of incorporating such additional assessments in future cases.
